# Improved survival after laparoscopic compared to open gastrectomy for advanced gastric cancer: a Swedish population-based cohort study

**DOI:** 10.1007/s10120-023-01371-8

**Published:** 2023-02-19

**Authors:** Andrianos Tsekrekos, Laura E. Vossen, Lars Lundell, Martin Jeremiasen, Erik Johnsson, Jakob Hedberg, David Edholm, Fredrik Klevebro, Magnus Nilsson, Ioannis Rouvelas

**Affiliations:** 1 Department of Upper Abdominal Surgery, Karolinska University Hospital, C1:77 Stockholm, Sweden; 2 Division of Surgery, Department of Clinical Science, Intervention and Technology (CLINTEC), Karolinska Institutet, Hälsovägen 13, 141 57, Huddinge, Stockholm, Sweden; 3 Centre for Bioinformatics and Biostatistics, Karolinska Institutet, Stockholm, Sweden; 4 Department of Surgery, Odense University Hospital, Odense, Denmark; 5 Department of Surgery, Skåne University Hospital and Department of Clinical Sciences, Lund University, Lund, Sweden; 6 Department of Surgery, Sahlgrenska University Hospital and Institute of Clinical Sciences, Sahlgrenska Academy at the University of Gothenburg, Gothenburg, Sweden; 7 Department of Surgical Sciences, Uppsala University, Uppsala, Sweden; 8 Department of Surgery, Biomedical and Clinical Sciences, Linköping University, Linköping, Sweden

**Keywords:** Advanced gastric cancer, Laparoscopic gastrectomy, Minimally invasive surgery, Survival

## Abstract

**Background:**

Laparoscopic gastrectomy is increasingly used for the treatment of locally advanced gastric cancer but concerns remain whether similar results can be obtained compared to open gastrectomy, especially in Western populations. This study compared the short-term postoperative, oncological and survival outcomes following laparoscopic versus open gastrectomy based on data from the Swedish National Register for Esophageal and Gastric Cancer.

**Methods:**

Patients who underwent surgery with curative intent for adenocarcinoma of the stomach or gastroesophageal junction Siewert type III from 2015 to 2020 were identified, and 622 patients with cT2-4aN0-3M0 tumors were included. The impact of surgical approach on short-term outcomes was assessed using multivariable logistic regression. Long-term survival was compared using multivariable Cox regression.

**Results:**

In total, 350 patients underwent open and 272 laparoscopic gastrectomy, of which 12.9% were converted to open surgery. The groups were similar regarding distribution of clinical disease stage (27.6% stage I, 46.0% stage II, and 26.4% stage III). Neoadjuvant chemotherapy was administered to 52.7% of the patients. There was no difference in the rate of postoperative complications, but laparoscopic approach was associated with lower 90 day mortality (1.8 vs 4.9%, *p* = 0.043). The median number of resected lymph nodes was higher after laparoscopic surgery (32 vs 26, *p* < 0.001), while no difference was found in the rate of tumor-free resection margins. Better overall survival was observed after laparoscopic gastrectomy (HR 0.63, *p* < 0.001).

**Conclusions:**

Laparoscopic gastrectomy can be safely preformed for advanced gastric cancer and is associated with improved overall survival compared to open surgery.

**Supplementary Information:**

The online version contains supplementary material available at 10.1007/s10120-023-01371-8.

## Introduction

Current guidelines on the treatment of locally advanced gastric cancer (AGC) recommend a combination of surgery and systemic oncological therapy [1–3]. The approach differs worldwide, and may involve perioperative chemotherapy [4–6], adjuvant chemotherapy [7–9], or chemoradiotherapy [10]. Irrespective of treatment strategy, radical tumor resection with complete regional lymphadenectomy (D2 lymph node dissection) remains the cornerstone of curative treatment.

Randomized controlled trials (RCT) conducted over the last decade have demonstrated several advantages of laparoscopic compared to open gastrectomy in AGC. Short-term benefits include reduced postoperative morbidity and earlier recovery, which usually also translate into shorter hospital stay [11–14]. Long-term outcomes have also been reported in recent years, showing that laparoscopic and open surgery are equivalent in terms of oncological safety and survival [15, 16]. The issue is that for the most part, the body of evidence on gastric cancer surgery is generated in high-incidence East Asian countries. Direct application to Western populations might not be appropriate, as several differences exist with regard to demographic factors and disease characteristics, that are likely to influence outcomes. In Europe, a higher proportion of patients are diagnosed with proximally located tumors and tumors of the poorly cohesive histological type, which require total gastrectomy. This, in combination with a higher age at diagnosis and a higher body mass index in average, as well as a different spectrum of—often obesity related—comorbidities, make surgery more challenging [17]. Important differences also exist in the standard treatment offered to patients with AGC, as for example, the extent to which neoadjuvant chemotherapy is utilized.

A few RCTs have been conducted in Europe and have confirmed the non-inferiority of laparoscopic surgery [18–20], but the generalizability of these results remains to be investigated in routine health care. The strict conditions mandated by the RCT design are not always reflecting the patient characteristics and surgical practices on a population level. Therefore, cohort studies based on population-based registers offer a valuable complement to RCTs. Given adequate coverage and data accuracy, such studies provide evidence of higher external validity [21, 22]. Sweden has a long tradition of developing and operating healthcare quality registers [23]. This study aimed to compare the outcomes after laparoscopic versus open surgery for AGC, based on data from a disease-specific register that covers the Swedish population.

## Materials and methods

The current study is reported following the recommendations of the Strengthening the Reporting of Observational Studies in Epidemiology (STROBE) initiative [24]. Ethical approval was obtained from the Regional Research Ethics Committee (EPN) of Stockholm (Dnr 2013/596–31/3 and 2016/1486–32).

### Data source

The study was conducted with data from the Swedish National Register for Esophageal and Gastric Cancer (NREV). The register was launched in 2006 and prospectively collects detailed information on all aspects of care for patients diagnosed with these malignancies in Sweden [25]. Perioperative data are acquired at three different time points and reported directly to NREV via online register software by the hospital responsible for the diagnosis, treatment and follow-up of the patient. The register has been described in detail elsewhere [26]. The NREV database has previously been validated and shown to have a data completeness rate of 95.5% and data accuracy of 91.1% [27]. Date of death was obtained from the Swedish population register.

### Study population

All patients who underwent surgery for adenocarcinoma of the stomach or gastroesophageal junction Siewert type III [28] between January 1st, 2015 and December 31st, 2020 were identified. Potentially eligible patients were considered for inclusion and baseline characteristics, details of the surgical procedure, and postoperative outcomes were extracted from the register database. Missing data (mainly owing to non-compliance with follow-up surveys) were supplemented by reviewing these patients’ electronic medical records. Outliers and possible discrepancies were also double-checked, and inaccurate values or misclassifications were corrected.

### Exposure

Patients were divided into two groups depending on surgical approach, i.e., laparoscopic gastrectomy (LG) and open gastrectomy (OG, reference group). All analyses were performed on an intention-to-treat basis (laparoscopic procedures converted to open were included in the LG group).

### Outcome measures

Postoperative complications were graded in severity according to the Clavien–Dindo (CD) classification [29] and analyzed as overall postoperative morbidity (defined as occurrence of any complication CD grade ≥ II) and incidence of severe complications (defined as CD grade ≥ III). All complications were recorded, but analysis was restricted to the main surgical complications (reoperation, anastomotic leakage, pancreatic fistula/pancreatitis), wound complications, and main non-surgical complications (cardiovascular, pulmonary, thromboembolic). Mortality within 30 and 90 days following surgery was calculated and overall survival (OS) analyzed. Patients were followed until death or the end of follow-up (January 2022), whichever occurred first. Finally, the study focused on pathological outcomes reflecting the oncological quality of surgery, such as the radicality of resection (tumor-free resection margins) and lymph node (LN) yield. In addition, adequate lymphadenectomy was defined as pathological analysis of ≥ 16 LNs in the specimen.

### Statistical analysis

Descriptive statistics were used to summarize and present baseline data. The Pearson’s *χ*^2^ or Fisher’s exact test were used for comparison of categorical variables and the Wilcoxon rank sum test for continuous variables. Tests were two-sided, with the level of significance set at 5%.

To assess the impact of the exposure of interest on the short-term outcomes (morbidity, mortality, adequacy of lymphadenectomy, tumor-free resection margins), two multivariable logistic regression models were constructed and odds ratios (OR) with corresponding 95% confidence interval (CI) were estimated. The main model incorporated the following predefined covariates: age, sex, American Society of Anesthesiologists (ASA) score, clinical stage according to the 8th edition of the UICC TNM Classification of Malignant Tumors [30], extent of gastrectomy (distal or total), and neoadjuvant treatment. An extended, exploratory model was also fitted, adding Eastern Cooperative Oncology Group (ECOG) performance status, body mass index (kg/m^2^), and year of surgery (grouped as 2015–2016, 2017–2018, and 2019–2020). The residuals were assessed to check for violation of the assumptions of normality, linearity and homoskedasticity. Influential values and outliers were visualized with diagnostic plots. In addition, the multivariable models were checked for collinearity among the covariates and goodness of fit (Hosmer–Lemeshow statistic). Diagnostic assessments of all the logistic regression models were satisfactory.

Median survival time was estimated by the Kaplan–Meier method and the survival curves were compared with the log-rank test. Survivors were censored at the last date the register database was assessed (January 2022). To examine the association between the exposure under investigation and survival, both univariable and multivariable analyses were performed using Cox proportional hazards regression. The estimated effect sizes are expressed as hazard ratios (HR) with associated 95% CI. The multivariable Cox models (main and exploratory) included the same covariates used in the logistic regression models as defined above, with the addition of tumor differentiation grade to the exploratory model. Furthermore, subgroup survival analyses based on extent of surgery (distal or total gastrectomy) were performed. The proportional hazards assumption was checked in all Cox models and, whenever a violation was found in the initial model, a stratified model was fitted to the data with stratification for the offending covariate(s).

All statistical analyses were performed using the R statistical software version 4.2.1 (R Foundation for Statistical Computing, Vienna, Austria) [31].

## Results

**Fig. 1:**
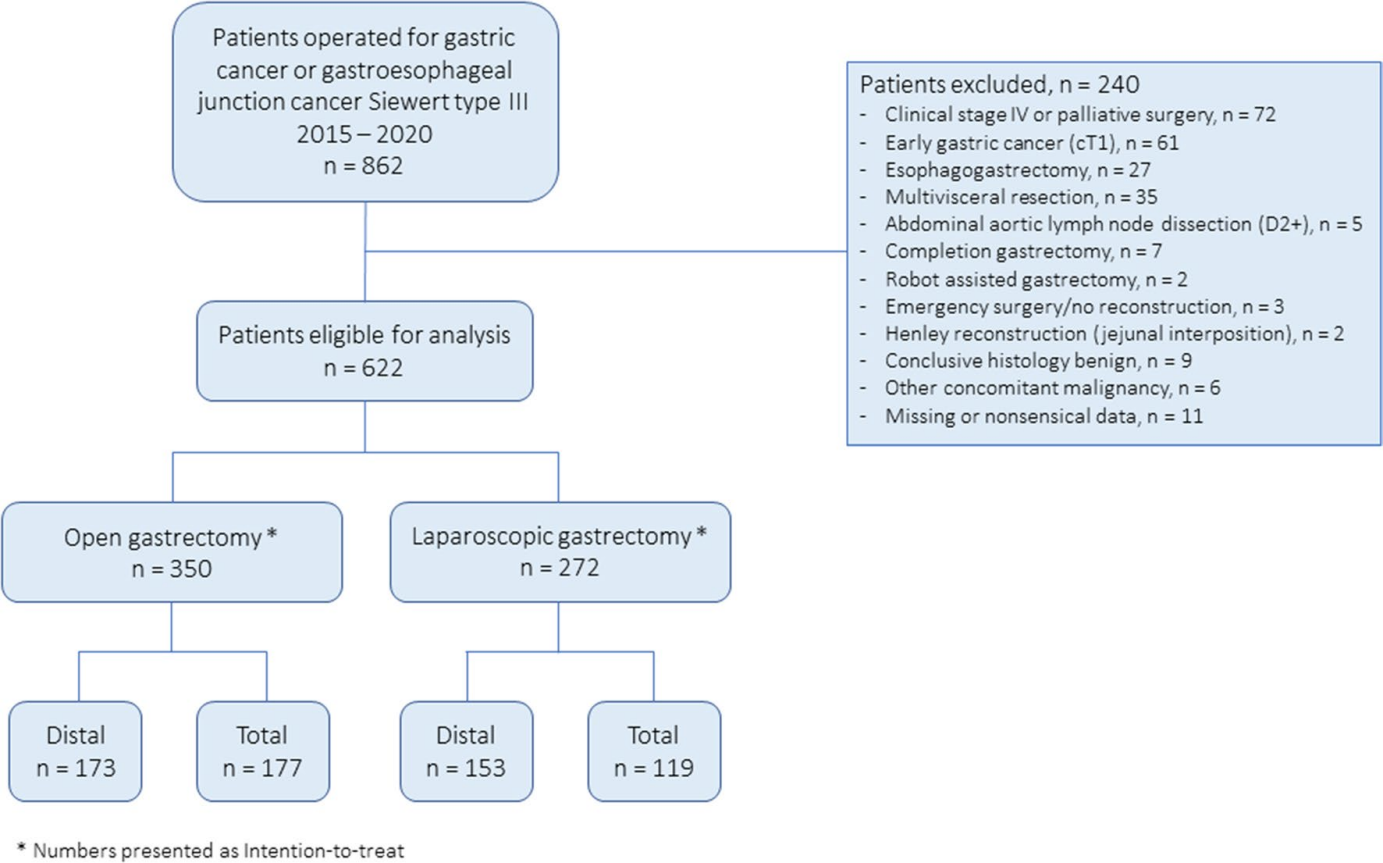
Flow chart of the selection of the study population

A total of 862 potentially eligible patients were identified during the study period. Two hundred and forty patients (27.8%) were eventually excluded from the study, based on the following criteria: early gastric cancer (*n* = 61), previous gastric surgery for benign or malignant disease (*n* = 7), tumor invading neighboring organ(s) mandating multivisceral resection beyond splenectomy (*n* = 35), tumor requiring combined esophagogastrectomy (*n* = 27), robot-assisted gastrectomy (*n* = 2), reconstruction with jejunal interposition (*n* = 2), emergency surgery without reconstruction (*n* = 3), extended lymphadenectomy (D2 +) including paraaortic LN dissection (*n* = 5), and palliative resection, or resection performed for clinical stage IV disease within the framework of clinical trials (*n* = 72). Further, patients were excluded in case of other concomitant malignancy at the time of diagnosis (*n* = 6). Finally, 9 patients were excluded because the conclusive pathological examination did not confirm gastric cancer, and 11 patients (1.7%) were excluded owing to missing or nonsensical data. As a result, 622 patients with cT2-4aN0-3M0 tumors that had undergone curative or borderline curative/palliative standard gastrectomy [2] were eligible for analysis. A flow chart of the selection of the study population is presented in Fig. 1.

### Patient characteristics

Baseline demographics, tumor characteristics and treatment details of the study population, as well as their distribution within the OG and LG groups, are summarized in Table 1. Of the 622 patients included, 258 (41.5%) were female. Mean age at the time of surgery was 68.9 years (range 26–90) and the mean BMI was 25.8 kg/m^2^ (range 14.8–48.0). The distribution of clinical disease stage in the study cohort was 27.6% stage I, 46.0% stage II, and 26.4% stage III. Neoadjuvant chemotherapy was administered to 52.7% of the patients. The treatment groups were well balanced at baseline, with the exception of tumor location in the stomach (more proximal tumors in the OG group) and year of surgery. Nevertheless, the extent of resection (distal or total gastrectomy) was similar between the groups (total gastrectomy, LG 43.8% vs OG 50.6%, *p* = 0.09). During the first 2 years of the study period, less than 30% of gastrectomies were performed laparoscopically, but from 2017 and onwards there was an approximately even distribution with respect to surgical approach and in 2019 laparoscopic procedures dominated.

**Table 1:** Demographics, tumor characteristics and treatment details of the study cohort

	Total	OG	LG	p*
	*n* = 622	*n* = 350	*n* = 272	
Age, years (mean ± SD)	68.9 ± 11.5	69.4 ± 11.2	68.2 ± 11.9	0.2
BMI, kg/m^2^ (mean ± SD)	25.8 ± 4.7	25.7 ± 4.8	25.9 ± 4.7	0.5
Sex				0.11
Male	364 (58.5)	195 (55.7)	169 (62.1)	
Female	258 (41.5)	155 (44.3)	103 (37.9)	
ASA score				0.7
1	165 (26.5)	97 (27.7)	68 (25.0)	
2	272 (43.7)	152 (43.4)	120 (44.1)	
3 +	185 (29.7)	101 (28.9)	84 (30.9)	
ECOG performance status				0.07
0	314 (50.5)	163 (46.6)	151 (55.5)	
1	215 (34.6)	133 (38.0)	82 (30.1)	
2 +	93 (14.9)	54 (15.4)	39 (14.3)	
Clinical T category				0.9
T2	197 (31.7)	111 (31.7)	86 (31.6)	
T3	332 (53.4)	189 (54.0)	143 (52.6)	
T4a	93 (15.0)	50 (14.3)	43 (15.8)	
Clinical N category				0.4
N0	433 (69.6)	246 (70.3)	187 (68.8)	
N1	111 (17.9)	62 (17.7)	49 (18.0)	
N2	58 (9.3)	28 (8.0)	30 (11.0)	
N3	20 (3.2)	14 (4.0)	6 (2.2)	
Clinical stage (TNM 8th edition [30])				0.3
I	172 (27.6)	93 (26.6)	79 (29.0)	
II	286 (46.0)	171 (48.9)	115 (42.3)	
III	164 (26.4)	86 (24.6)	78 (28.7)	
Tumor location				**0.02**
Pylorus	91 (14.6)	48 (13.7)	43 (15.8)	
Antrum	202 (32.5)	98 (28.0)	104 (38.2)	
Corpus	224 (36.0)	136 (38.9)	88 (32.4)	
Fundus	32 (5.1)	23 (6.6)	9 (3.3)	
Cardia type III	49 (7.9)	33 (9.4)	16 (5.9)	
Unspecified or multiple locations	24 (3.9)	12 (3.4)	12 (4.4)	
Type of gastrectomy				0.09
Distal	326 (52.4)	173 (49.4)	153 (56.2)	
Total	296 (47.6)	177 (50.6)	119 (43.8)	
Splenectomy performed	32 (5.1)	22 (6.3)	10 (3.7)	0.14
Neoadjuvant treatment				0.2
None	294 (47.3)	173 (49.4)	121 (44.5)	
Chemotherapy	328 (52.7)	177 (50.6)	151 (55.5)	
Calendar year of surgery				** < 0.001**
2015	80 (12.9)	58 (72.5)	22 (27.5)	
2016	107 (17.2)	78 (72.9)	29 (27.1)	
2017	97 (15.6)	49 (50.5)	48 (49.5)	
2018	123 (19.8)	62 (50.4)	61 (49.6)	
2019	117 (18.8)	52 (44.4)	65 (55.6)	
2020	98 (15.8)	51 (52.0)	47 (48.0)	

*OG* open gastrectomy, *LG* laparoscopic gastrectomy, *SD* standard deviation, *BMI* body mass index, *ASA* American Society of Anesthesiologists, *ECOG* Eastern Cooperative Oncology Group

Data presented as *n* (%), unless otherwise indicated

Percentages may not add up to 100% because of rounding

*Wilcoxon rank sum test for continuous variables. Pearson’s *χ*^2^ test for categorical variables with all expected cell counts ≥ 5. Fisher’s Exact test for categorical variables with any expected cell count < 5. Significant values (*p* < 0.05) are indicated with bold characters

### Operative results and short-term postoperative outcomes

Three hundred and fifty patients underwent OG and 272 patients LG, of which 35 (12.9%) were converted to open surgery. Overall, 326 patients (52.4%) underwent distal gastrectomy and 296 (47.6%) total gastrectomy. Splenectomy was performed in 5.1% of the cases (LG 3.7% vs OG 6.3%, *p* = 0.14). LG was associated with longer operating time [median 300 min (IQR 214–375) vs 209 min (IQR 157–280), *p* < 0.001], and less blood loss [median 100 mL (IQR 50–200) vs 250 mL (IQR 150–500), *p* < 0.001]. Length of hospital stay was 1 day shorter in the LG group, a difference that was not significant (Table 2).

**Table 2:** Operative results and postoperative outcomes by surgical approach

	Total	OG	LG	p*
	*n* = 622	*n* = 350	*n* = 272	
Conversion to open surgery	–	–	35 (12.9)	
Median operating time, min (IQR)	240 (177–330)	209 (157–280)	300 (214–375)	** < 0.001**
Median estimated blood loss, mL (IQR)	200 (100–400)	250 (150–500)	100 (50–200)	** < 0.001**
Overall complications–CD grade ≥ II	232 (37.3)	131 (37.4)	101 (37.1)	> 0.9
II	109 (17.5)	58 (16.6)	51 (18.8)	
IIIa	36 (5.8)	23 (6.6)	13 (4.8)	
IIIb	52 (8.4)	28 (8.0)	24 (8.8)	
IVa	15 (2.4)	7 (2.0)	8 (2.9)	
IVb	7 (1.1)	5 (1.4)	2 (0.7)	
V	13 (2.1)	10 (2.9)	3 (1.1)	
Severe complications—CD grade ≥ III	123 (19.8)	73 (20.9)	50 (18.4)	0.4
ICU admission	30 (4.8)	17 (4.9)	13 (4.8)	> 0.9
Reoperation/Intervention	63 (10.1)	33 (9.4)	30 (11.0)	0.5
Surgical complications	125 (20.1)	69 (19.7)	56 (20.6)	0.8
Intraabdominal complications	113 (18.2)	61 (17.4)	52 (19.1)	0.6
Anastomotic leakage	45 (7.2)	27 (7.7)	18 (6.6)	
Intraabdominal abscess	34 (5.5)	19 (5.4)	15 (5.5)	
Bleeding	13 (2.1)	8 (2.3)	5 (1.8)	
Pancreatitis/pancreatic fistula	11 (1.8)	5 (1.4)	6 (2.2)	
Small bowel obstruction	22 (3.5)	8 (2.3)	14 (5.1)	
Bowel perforation	7 (1.1)	4 (1.1)	3 (1.1)	
Wound complications	20 (3.2)	14 (4.0)	6 (2.2)	0.2
Infection/abscess	16 (2.6)	10 (2.9)	6 (2.2)	
Fascia dehiscence	8 (1.3)	7 (2.0)	1 (0.4)	
Other infectious complication	54 (8.7)	29 (8.3)	25 (9.2)	0.7
Non-surgical complications	102 (16.4)	54 (15.4)	48 (17.6)	0.5
Cardiovascular	24 (3.9)	15 (4.3)	9 (3.3)	0.5
Pulmonary	75 (12.1)	38 (10.9)	37 (13.6)	0.3
Thromboembolic	17 (2.7)	11 (3.1)	6 (2.2)	0.5
Other complications	64 (10.3)	42 (12.0)	22 (8.1)	0.11
Median hospital stay, days (IQR)	8 (6–12)	8 (6–12)	7 (6–12)	0.3
Postoperative mortality				
30 day	10 (1.6)	9 (2.6)	1 (0.4)	**0.049**
90 day	22 (3.5)	17 (4.9)	5 (1.8)	**0.043**

*OG* open gastrectomy, *LG* laparoscopic gastrectomy, *IQR* interquartile range, *CD* Clavien–Dindo, *ICU* intensive care unit

Data presented as *n* (%), unless otherwise indicated

Percentages may not add up to 100% because of rounding

*Wilcoxon rank sum test for continuous variables. Pearson’s *χ*^2^ test for categorical variables with all expected cell counts ≥ 5. Fisher’s Exact test for categorical variables with any expected cell count < 5. Significant values (*p* < 0.05) are indicated with bold characters

There was no difference in the rate of overall postoperative complications (LG 37.1% vs OG 37.4%, *p* > 0.9), or severe complications (LG 18.4% vs OG 20.9%, *p* = 0.4). In the main multivariable logistic regression analysis, surgical approach was not associated with the occurrence of postoperative complications (Supplementary Table 1). Similar results were obtained in the exploratory model, with no significant alterations in the ORs (data not shown).

A lower mortality was recorded in the LG group, both 30 days (0.4% vs 2.6%, *p* = 0.049) and 90 days after surgery (1.8% vs 4.9%, *p* = 0.043). Multivariable regression analysis confirmed this finding, with lower 30 day mortality (adjusted OR 0.13, 95% CI 0.01–0.75, *p* = 0.06) and 90 day mortality (adjusted OR 0.38, 95% CI 0.12–0.99, *p* = 0.06) in favor of LG.

### Pathological findings

The groups were different with regard to tumor differentiation grade, with more well differentiated (G1) tumors in the LG group and a higher proportion of moderately differentiated (G2) tumors in the OG group. The proportion of poorly differentiated (G3) tumors was similar between the groups. The two groups were also comparable regarding pathological TNM stage, with the exception of pN-status; pN0 was a more common finding in the LG group, while pN3 cases were overrepresented in the OG group. There was no difference in the observed complete tumor regression rate (LG 4.0% vs OG 5.1%, *p* = 0.5). The median number of resected LNs was higher in the LG group [32 (IQR 23–47) vs 26 (IQR 18–33), *p* < 0.001], as was the proportion of patients that had an adequate lymphadenectomy, i.e., a minimum of 16 LNs removed (91.2% vs 81.7%, *p* < 0.001) (Table 3). Multivariable logistic regression identified the laparoscopic approach as an independent predictor of adequate lymphadenectomy (adjusted OR 2.94, 95% CI 1.75–5.13, *p* < 0.001). In contrast, no difference was found with regard to the radicality of surgery (microscopically tumor-free resection margins, R0) between the study groups (adjusted OR 1.00, 95% CI 0.56–1.75, *p* = 0.99).

**Table 3:** Pathological findings by surgical approach

	Total	OG	LG	p*
	*n* = 622	*n* = 350	*n* = 272	
Tumor differentiation				** < 0.001**
G1—well differentiated	95 (15.3)	26 (7.4)	69 (25.4)	
G2—moderately differentiated	89 (14.3)	62 (17.7)	27 (9.9)	
G3—poorly differentiated	339 (54.5)	197 (56.3)	142 (52.2)	
Undifferentiated	15 (2.4)	13 (3.7)	2 (0.7)	
Not assessed/specified	55 (8.8)	34 (9.7)	21 (7.7)	
Complete tumor regression	29 (4.7)	18 (5.1)	11 (4.0)	0.5
pT stage				0.2
HGD	5 (0.8)	1 (0.3)	4 (1.5)	
T1	96 (15.4)	49 (14.0)	47 (17.3)	
T2	95 (15.3)	48 (13.7)	47 (17.3)	
T3	188 (30.2)	106 (30.3)	82 (30.1)	
T4	209 (33.6)	128 (36.6)	81 (29.8)	
pN stage				**0.006**
N0	267 (42.9)	135 (38.6)	132 (48.5)	
N1	97 (15.6)	51 (14.6)	46 (16.9)	
N2	106 (17.0)	61 (17.4)	45 (16.5)	
N3	152 (24.4)	103 (29.4)	49 (18.0)	
pM stage				0.08
M0	599 (96.3)	333 (95.1)	266 (97.8)	
M1	23 (3.7)	17 (4.9)	6 (2.2)	
Resected lymph nodes, median (IQR)	27 (20–39)	26 (18–33)	32 (23–47)	** < 0.001**
≥ 16 lymph nodes retrieved	534 (85.9)	286 (81.7)	248 (91.2)	** < 0.001**
Radicality				0.6
R0	563 (90.5)	315 (90.0)	248 (91.2)	
R1	56 (9.0)	34 (9.7)	22 (8.1)	
Rx	3 (0.5)	1 (0.3)	2 (0.7)	

*OG* open gastrectomy, *LG* laparoscopic gastrectomy, *HGD* high-grade dysplasia, *IQR* interquartile range, *Rx* equals unscertain radicality

Data presented as n (%), unless otherwise indicated

Percentages may not add up to 100% because of rounding

*Wilcoxon rank sum test for continuous variables. Pearson’s *χ*^2^ test for categorical variables with all expected cell counts ≥ 5. Fisher’s Exact test for categorical variables with any expected cell count < 5. Significant values (*p* < 0.05) are indicated with bold characters

### Overall survival

**Fig. 2:**
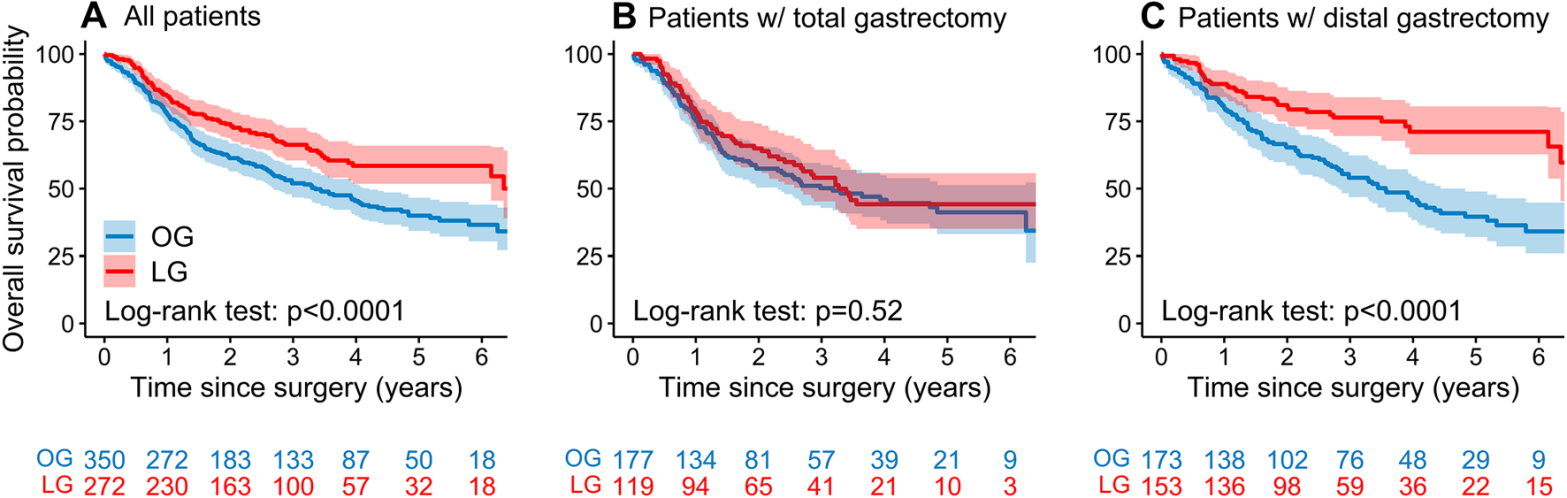
Kaplan–Meier estimates of overall survival by surgical approach (laparoscopic vs open gastrectomy). (A) All patients, (B) Patients operated with total gastrectomy and (C) Patients operated with distal gastrectomy. *OG* open gastrectomy, *LG* laparoscopic gastrectomy

The median follow-up time of the cohort was 49.2 months, with no patients lost to follow-up. Overall survival was better after LG, with the median survival time not reached, compared to 40.2 months following OG. The estimated 5 year survival rates were 58 and 40%, respectively. Subgroup analysis revealed that the survival benefit was seen exclusively among patients who underwent distal gastrectomy (Fig. 2). In the multivariable Cox regression analysis LG was associated with an HR of 0.63 (95% CI 0.49–0.81, *p* < 0.001). In the subgroup analysis, the improvement in OS remained among the patients who underwent distal gastrectomy, while no difference was demonstrated in the total gastrectomy group (Table 4). The exploratory model showed similar results, although here there was a less pronounced benefit of the laparoscopic approach, with HR 0.73 (95% 0.56–0.95, *p* = 0.02).

**Table 4:** Crude and adjusted hazard ratio estimates for overall survival by surgical approach

	Crude HR (95% CI)	*p*	Adjusted ^a^ HR (95% CI)	*p*	Adjusted ^b^ HR (95% CI)	*p*
All gastrectomies						
Open	1.00 (reference)		1.00 (reference)		1.00 (reference)	
Laparoscopic	**0.63 (0.49–0.81)**	** < 0.001**	**0.63 (0.49–0.81)**	** < 0.001**	**0.73 (0.56–0.95)**	**0.02**
Distal gastrectomies						
Open	1.00 (reference)		1.00 (reference)		1.00 (reference)	
Laparoscopic	**0.44 (0.30–0.64)**	** < 0.001**	**0.51 (0.35–0.74)**	** < 0.001**	0.69 (0.45**–**1.05)	0.09
Total gastrectomies						
Open	1.00 (reference)		1.00 (reference)		1.00 (reference)	
Laparoscopic	0.90 (0.65**–**1.25)	0.5	0.87 (0.58**–**1.30)	0.5	0.88 (0.61**–**1.28)	0.5

*HR* hazard ratio, *CI* confidence interval

Multivariable Cox proportional hazards regression model adjusted for:

^a^age, sex, ASA (American Society of Anesthesiologists) score, clinical TNM stage, neoadjuvant treatment (main model)

^b^age, sex, ASA score, clinical TNM stage, neoadjuvant treatment, ECOG (Eastern Cooperative Oncology Group) performance status, BMI (body mass index), tumor differentiation grade and year of surgery (exploratory model)

Significant values are indicated with bold characters

## Discussion

The main findings of this population-based study suggest that LG for AGC is associated with improved OS compared to OG. The estimated survival benefit remained significant after adjustment for a number of patient- and treatment-related factors that are known to affect long-term survival. We could also demonstrate that LG is an oncologically safe procedure for AGC, based on the assessment of a number of parameters that are commonly used to reflect the quality of gastric cancer surgery. We observed similar postoperative morbidity irrespective of surgical approach, while LG exhibited a lower 30 and 90 day mortality compared to OG.

The question whether LG for AGC offers comparable results to OG in terms of long-term survival has been investigated mainly in East Asian countries, where the disease is considerably more prevalent. A multicenter cohort study from Japan, including 610 patients of which 40% underwent total gastrectomy, showed similar survival rates between LG and OG [32]. Two large RCTs from China and Korea, with approximately 1000 patients each, have subsequently confirmed the non-inferiority of LG, reporting a 5 year OS similar to that obtained after OG [15, 16]. Those randomized trials were restricted to patients undergoing distal gastrectomy and, as is usually the case with RCTs, several other specific patient entry criteria were determined that limit their generalizability. As an example, patients older than 80 years of age were excluded from those trials. In our cohort, this patient group comprised approximately 17% of the study population. Similarly, ECOG PS > 1 and ASA score > 3 were additional reasons for patient exclusion. Based on our data, another 10% of patients undergoing gastrectomy in Sweden would fall into one of those categories. Even more important, no neoadjuvant chemotherapy is routinely administered in Asia, which was the case for half of the patients in the present study. Recently, the Italian Research Group for Gastric Cancer conducted a multicenter study comparing LG and OG for AGC on a propensity score matched cohort (where 24% of patients received neoadjuvant chemotherapy and 36% underwent total gastrectomy), showing no difference in 3 year OS [33].

Our results also indicate that the pathological criteria reflecting the oncological quality of the procedure can be met by the laparoscopic approach. Similar rates of radical resection were observed between LG and OG (91.2 and 90.0% respectively), while LG resulted in a significantly higher number of retrieved LNs, with a median of 32 compared to 26 LNs following OG. In the LG group, 91.2% of the patients had at least 16 LNs removed, compared to 81.7% in the OG group. We used the threshold of 16 LNs since it is considered the minimum for a reliable pathological staging in both the latest UICC TNM classification [30] and the most recent Japanese recommendations [34]. The multivariable analysis identified LG as an independent predictor of adequate lymphadenectomy, associated with an OR of 2.94. These favorable results are in agreement with prospectively collected data from Asia, as well as the STOMACH trial from Europe [11–13, 19].

Furthermore, we found that the occurrence of overall postoperative complications and severe complications, including anastomotic leakage, did not differ between the treatment groups. It is important to interpret those results in light of the fact that the early years of implementation of the laparoscopic technique were not excluded from the analysis, meaning that they incorporate the learning curve of the procedure [35, 36]. A population-based study from the Netherlands came to the same conclusions [37]. One of the important features of our study population was that just over 50% of the patients received chemotherapy before surgery. Since neoadjuvant chemotherapy is not part of standard treatment protocols in countries with high incidence of gastric cancer, evidence is limited and the role of laparoscopic surgery in this context is not yet clarified. The LOGICA trial [20] included patients with predominantly AGC (76%), of which 72% were treated with neoadjuvant chemotherapy. Similar to our findings, LG and OG did not differ with regard to postoperative complications. Added to the two aforementioned European trials [19, 20], there is one phase II trial from China that has investigated the safety of laparoscopic distal gastrectomy following neoadjuvant chemotherapy and which also demonstrated no negative impact of chemotherapy on the surgical outcomes [14].

Our primary finding was that LG, in particular distal gastrectomy, is associated with better OS, but the reason for that is not clear. The currently observed differences in postoperative mortality and long-term survival cannot be explained by differences in the occurrence of severe complications requiring invasive intervention or ICU admission. On the other hand, diminishing the surgical trauma by performing a less invasive procedure will spare the patient’s physiological reserves and enhance the ability to cope with severe complications that may occur. Studies investigating the immunological response following laparoscopic gastrectomy have revealed lower IL-6 and C-reactive protein levels compared to open surgery, implying less impact on the immune system. The fact that immune function is better preserved after minimally invasive surgery may have contributed to the lower postoperative mortality observed [38]. It can also be argued that the higher LN yield may play a role, since it is recognized that D2 lymphadenectomy confers a survival benefit in Western populations as well [39, 40]. Nevertheless, a subgroup analysis showed that the number of resected LNs was significantly higher after both distal and total LG, compared to the open counterparts (data not shown). One possible explanation for the difference in survival would be a corresponding difference in relapse rate. It should be noted that, although the treatment groups were well balanced in terms of clinical staging, a higher frequency of node-positive disease was subsequently found in the OG group. Unfortunately, information on disease recurrence is not available in the Swedish register. Gastric cancer is a heterogeneous malignancy where response to treatment can vary considerably. Molecular classification of gastric cancer, linked to distinct genomic alterations, has defined four major subtypes that have an impact on survival and recurrence patterns [41]. The microsatellite-unstable tumors, having the best prognosis, are mainly of the intestinal type, located predominantly in the antrum, and thus amenable to distal gastrectomy. On the opposite side of the spectrum is the mesenchymal-like subtype (including diffuse gastric cancer), with the worst prognosis and highest risk for relapse. This type of gastric cancer almost always requires total gastrectomy to achieve a radical resection. Differences in tumor biology and aggressiveness may explain the finding that no survival benefit was observed in the group of patients requiring total gastrectomy. Finally, one important aspect of the multimodal treatment for AGC is the extent to which patients are able to tolerate and complete the preplanned adjuvant chemotherapy. A number of studies have highlighted that the laparoscopic approach may result in higher rates of administration of the intended systemic therapy after gastrectomy [14, 33, 42, 43]. This is a very important observation, and a potential positive effect of minimally invasive surgery is worth investigating in dedicated future studies.

There are certain limitations in our study that need to be acknowledged. Being a register-based study and retrospective in nature, any adjustment for possible confounding factors was restricted by the variables available in the register. Baseline characteristics that certainly affect outcomes, such as nutritional status or co-morbidities measured on a validated scale, were not recorded during the first years of the study period. Still, we were able to adjust for ASA score and ECOG PS, which can be considered to sum up the patient’s functional status and burden of associated co-morbidities. The non-surgical component of the treatment is solely reported as the intention to proceed with chemotherapy, thus we lack information on whether adjuvant chemotherapy was ultimately administered as intended. Completion of adjuvant chemotherapy is without doubt of importance when assessing survival, but it was not possible to adjust for in our model. Likewise, information on the implementation of Enhanced Recovery Programs (ERPs) was unavailable and a possible confounding effect could not be excluded. This is a potential source of residual confounding, as ERPs may have been introduced earlier in teaching hospitals, which were also early on with implementing minimally invasive surgery for AGC. The main strength of this study is the large sample size from a Western perspective, which is population-based and unselected. Much effort was put into minimizing the amount of missing data by reviewing medical records as necessary, increasing data completeness to 98%. Only patients with complete data on all variables were included in the final analysis, with no loss to follow-up, and a proper adjustment for confounding factors was performed.

## Conclusions

Our findings imply that LG can be safely preformed for AGC and is may be associated with improved OS compared to OG in a Western population. The current study adds to the existing evidence supporting the adoption of LG as standard treatment for AGC.

## Supplementary Information

Below is the link to the electronic supplementary material.

Supplementary file1 (PDF 141 KB)

## Data Availability

The data in this study are available from the corresponding author upon reasonable request and given that approvals are obtained from the relevant research ethics committee(s) and the authority legally responsible for the handling of personal data, in compliance with the General Data Protection Regulation (GDPR) requirements.
