# Supplementary material for: Improved survival after laparoscopic compared to open gastrectomy for advanced gastric cancer: a Swedish population-based cohort study
Source: Gastric Cancer. 2023 Feb 19;26(3):467–77. doi: 10.1007/s10120-023-01371-8 (PMC10115725; doi:10.1007/s10120-023-01371-8)
Supplement: Supplementary file 1 — Supplementary file1 (PDF 141 KB) [file 10120_2023_1371_MOESM1_ESM.pdf]

Gastric Cancer

Improved survival after laparoscopic compared to open gastrectomy for advanced gastric cancer: a Swedish population-based cohort study

Andrianos Tsekrekos MD, Laura E. Vossen PhD, Lars Lundell MD, PhD, Martin Jeremiasen MD, PhD, Erik Johnsson MD, PhD, Jakob Hedberg MD, PhD, David Edholm, MD, PhD, Fredrik Klevebro MD, PhD, Magnus Nilsson MD, PhD, Ioannis Rouvelas MD, PhD

Corresponding author:
Andrianos Tsekrekos
Department of Upper Abdominal Surgery, Karolinska University Hospital & Division of Surgery, Department of Clinical Science, Intervention and Technology (CLINTEC), Karolinska Institutet
Hälsovägen 13, 141 57 Huddinge, Stockholm, Sweden
E-mail address: andrianos.tsekrekos@ki.se

Supplementary Table 1 Crude and adjusted odds ratio estimates for postoperative complications*

	OG<br>OR	LG<br>OR (95% CI)	*p*
Overall complications – CD grade ≥II			
Crude	1.00 (reference)	0.99 (0.71 – 1.37)	0.94
Adjusted	1.00 (reference)	1.07 (0.76 – 1.52)	0.68
Severe complications – CD grade ≥III			
Crude	1.00 (reference)	0.85 (0.57 – 1.27)	0.44
Adjusted	1.00 (reference)	0.93 (0.61 – 1.42)	0.75
Surgical complications			
Crude	1.00 (reference)	1.06 (0.71 – 1.57)	0.79
Adjusted	1.00 (reference)	1.15 (0.76 – 1.74)	0.51

Reoperation			
Crude	1.00 (reference)	1.19 (0.70 – 2.01)	0.51
Adjusted	1.00 (reference)	1.31 (0.76 – 2.25)	0.32
Anastomotic leakage			
Crude	1.00 (reference)	0.85 (0.45 – 1.56)	0.60
Adjusted	1.00 (reference)	1.00 (0.51 – 1.90)	1.00
Pancreatic fistula/pancreatitis			
Crude	1.00 (reference)	1.56 (0.46 – 5.45)	0.47
Adjusted	1.00 (reference)	2.06 (0.57 – 7.89)	0.27
Wound complications			
Crude	1.00 (reference)	0.54 (0.19 – 1.37)	0.21
Adjusted	1.00 (reference)	0.53 (0.18 – 1.37)	0.21
Non-surgical complications			
Crude	1.00 (reference)	1.17 (0.77 – 1.80)	0.46
Adjusted	1.00 (reference)	1.25 (0.80 – 1.95)	0.33
Cardiovascular			
Crude	1.00 (reference)	0.76 (0.32 – 1.74)	0.53
Adjusted	1.00 (reference)	0.77 (0.31 – 1.81)	0.55
Pulmonary			
Crude	1.00 (reference)	1.29 (0.80 – 2.10)	0.30
Adjusted	1.00 (reference)	1.40 (0.84 – 2.34)	0.20
Thromboembolic			
Crude	1.00 (reference)	0.70 (0.24 – 1.85)	0.48
Adjusted	1.00 (reference)	0.73 (0.24 – 1.98)	0.54

OG open gastrectomy, *LG* laparoscopic gastrectomy, *OR* odds ratio, *CI* confidence interval, *CD* Clavien-Dindo

* Univariable logistic regression and multivariable logistic regression model adjusted for age, sex, ASA (American Society of Anesthesiologists) score, clinical TNM stage, extent of gastrectomy and neoadjuvant treatment.
